# Small RNAs in vancomycin-resistant Enterococcus faecium involved in daptomycin response and resistance

**DOI:** 10.1038/s41598-017-11265-2

**Published:** 2017-09-11

**Authors:** Clara Sinel, Yoann Augagneur, Mohamed Sassi, Julie Bronsard, Margherita Cacaci, François Guérin, Maurizio Sanguinetti, Pierrick Meignen, Vincent Cattoir, Brice Felden

**Affiliations:** 1 University of Caen Normandie, EA4655 Caen, France; 2 Inserm U1230-Biochimie pharmaceutique, Rennes University, Rennes, France; 3 Catholic University of Sacred Heart, Institute of Microbiology, Rome, Italy; 4 Caen University Hospital, Department of Clinical Microbiology, Caen, France; 5 University of Caen Normandie, IUT (department “STID”), Caen, France; 6 National Reference Center for Antimicrobial Resistance (lab Enterococci), Caen, France; 7 Present Address: Inserm U1230-Biochimie pharmaceutique, Rennes University, Rennes, France

## Abstract

Vancomycin-resistant *Enterococcus faecium* is a leading cause of hospital-acquired infections and outbreaks. Regulatory RNAs (sRNAs) are major players in adaptive responses, including antibiotic resistance. They were extensively studied in gram-negative bacteria, but less information is available for gram-positive pathogens. No sRNAs are described in *E*. *faecium*. We sought to identify a set of sRNAs expressed in vancomycin-resistant *E*. *faecium* Aus0004 strain to assess their roles in daptomycin response and resistance. Genomic and transcriptomic analyses revealed a set of 61 sRNA candidates, including 10 that were further tested and validated by Northern and qPCR. RNA-seq was performed with and without subinhibitory concentrations (SICs) of daptomycin, an antibiotic used to treat enterococcal infections. After daptomycin SIC exposure, the expression of 260 coding and srna genes was altered, with 80 upregulated and 180 downregulated, including 51% involved in carbohydrate and transport metabolisms. Daptomycin SIC exposure significantly affected the expression of seven sRNAs, including one experimentally confirmed, sRNA_0160. We studied sRNA expression in isogenic mutants with increasing levels of daptomycin resistance and observed that expression of several sRNAs, including sRNA_0160, was modified in the stepwise mutants. This first genome-wide sRNA identification in *E*. *faecium* suggests that some sRNAs are linked to antibiotic stress response and resistance.

## Introduction

Enterococci are commensals of the gastrointestinal microbiota of many animal species^1^. Within the genus, *Enterococcus faecalis* and *Enterococcus faecium* have emerged as major opportunistic pathogens^2^. They have become resistant to numerous antibiotics^3^, with the spread of vancomycin-resistant enterococci (VRE), especially in *E*. *faecium* ^4^. The latter is part of the ESKAPE (***E***. *faecium*, ***S*** *taphylococcus aureus*, ***K*** *lebsiella pneumoniae*, ***A*** *cinetobacter baumannii*, ***P*** *seudomonas aeruginosa* and ***E*** *nterobacter* spp.) group of major multidrug-resistant (MDR) nosocomial pathogens^5^. *E*. *faecium* antimicrobial resistance is worrisome because of the dissemination of hospital-adapted clones belonging to the clonal complex 17 (CC17)^6^. Epidemic CC17 strains are part of a human hospital-adapted lineage (clade A1) that emerged from the animal-associated lineage (clade A2) after the introduction of antibiotics, and which differs genetically from the human community-associated lineage (clade B)^7^. Thanks to its huge genomic plasticity and metabolic versatility, *E*. *faecium* is a highly adapted commensal bacterial species that can turn into an opportunistic pathogen^8^. Despite their paramount importance, the mechanisms involved in this physiological transition have not been adequately investigated^9^.

Among the numerous environmental stresses with which bacteria must cope to survive, the presence of antibiotics, especially at subinhibitory concentrations (SICs, concentrations below the MIC, which do not significantly affect bacterial growth), is suspected to play a key role in the origin and evolution of antimicrobial resistance^10^. SICs are expected to occur during antibiotic treatment in humans, when drug diffusion at the infection site is inadequate or during exposure of the gastrointestinal tract microbiota to antibiotics^11^. Interestingly, a recent study has demonstrated that exposure to ciprofloxacin SICs enhance antimicrobial resistance and pathogenicity in *E*. *faecium* ^12^. Cyclic lipopeptide daptomycin is a commonly used antibiotic to treat vancomycin-resistant *E*. *faecium* (VREF) infections^4^, via a mechanism involving calcium-dependent interaction with the bacterial membrane that modifies its integrity and leads to cell death^13^. Daptomycin resistance in *E*. *faecium* is still rare, but treatment failures are increasingly reported, related to the emergence of high-level daptomycin resistance^14, 15^. Although several genes (e.g. *liaFSR*, *yycFGHIJ*, and *cls*) are known to be involved in the development of daptomycin resistance^16^, the different steps of resistance acquisition remain unclear, particularly those related to the selection of low-level resistant mutants^17^. Moreover, nothing is currently known about the stress response of *E*. *faecium* to daptomycin exposure.

In recent years, several studies have shown that antibiotic exposure is correlated with the expression of bacterial regulatory small RNAs (sRNA)^18^. These sRNAs are usually short (50–600 nts) noncoding transcripts synthesized under specific environmental conditions. Modulating the expression level of target genes, mainly post-transcription, to enable rapid, tight adaptation to cellular physiology, which includes pathogenicity and antimicrobial resistance^19^, they can either enhance resistance (*e*.*g*. MicF in *E*. *coli* ^20^) or increase susceptibility (*e*.*g*. SprX in *S*. *aureus* ^21^) to antibacterial agents. Recent in-depth transcriptomic analyses of several bacterial species, in the presence of antibiotic SICs, have revealed that antibiotic exposure significantly modifies the expression of numerous sRNAs^22, 23^.

Although many sRNAs have been identified in *E*. *faecalis* ^24, 25^, nothing is currently known about the *E*. *faecium* sRNome. This lack of knowledge is particularly detrimental because the worldwide ratio of *E*. *faecalis*/*E*. *faecium* infections is currently changing in favor of *E*. *faecium* ^4^. To search for sRNAs expressed in *E*. *faecium*, we performed a genome-wide transcriptome analysis (RNA-seq) onto a hospital-adapted CC17 VREF clinical isolate^26^. Using the DETR’PROK workflow, a pipeline devoted to sRNA identification in prokaryotes, combined with an *in silico* search for the conserved sRNAs in bacteria and RNA-seq depth testing within the intergenic regions (IGRs), we identified 61 sRNA candidates. Then we monitored the levels of all transcripts in bacterial cells grown with and without daptomycin SICs. Finally, we studied the transcript levels of these sRNAs in a series of isogenic mutants, with increasing levels of daptomycin resistance. Our study demonstrates that *E*. *faecium* expresses many sRNAs and that the expression of several of them is induced or repressed by antibiotic exposure or during development of resistance. Our finding that the expression of sRNA_0160 is downregulated by daptomycin exposure and repressed in daptomycin-resistant mutants implies that it is connected to antibiotic response and resistance in *E*. *faecium*. Accordingly, we plan to explore its function and molecular targets in the future.

## Results

### Identification of sRNAs in *Enterococcus faecium*

**Figure 1:**
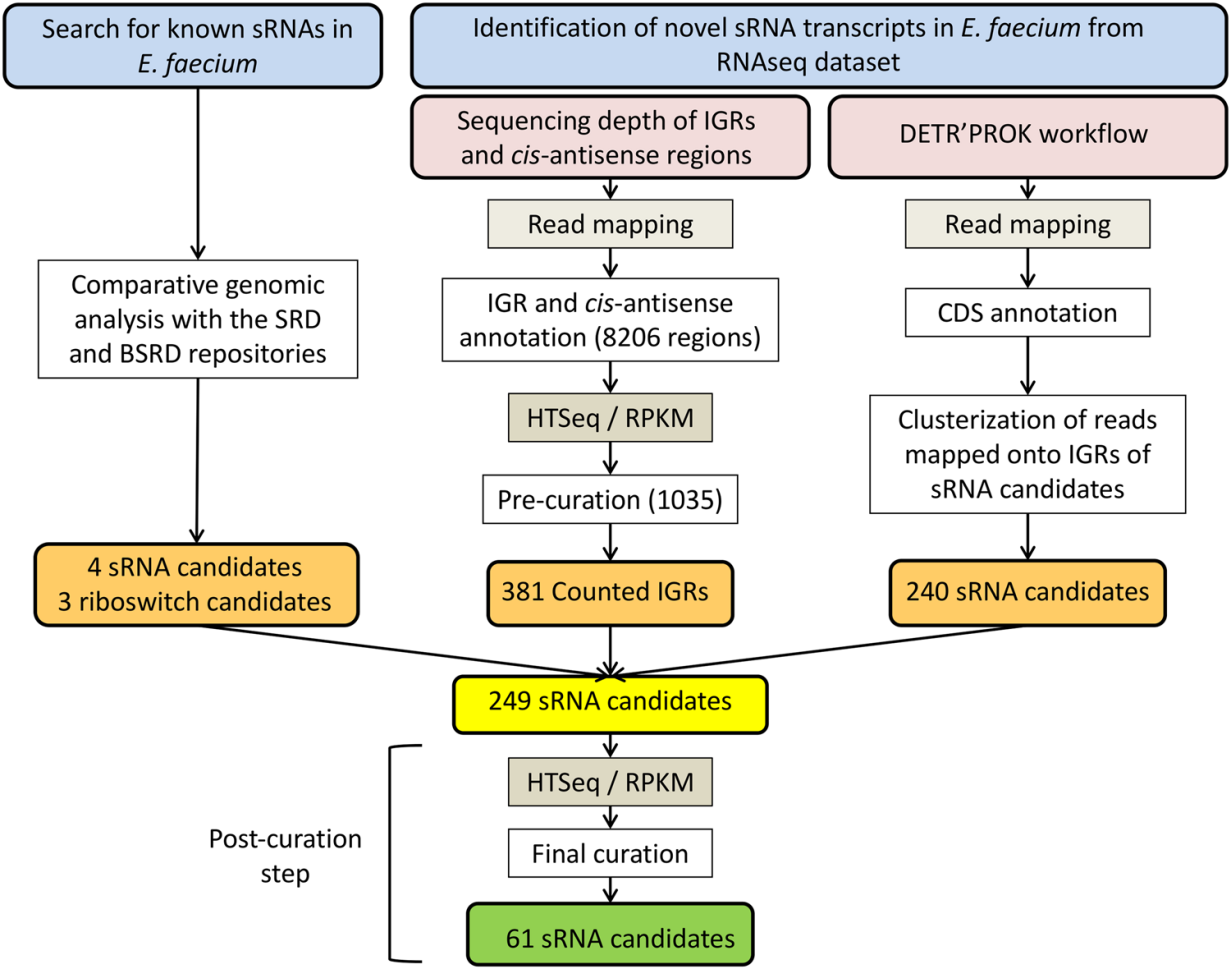
Identification of sRNA candidates in *Enterococcus faecium*. sRNAs were identified either by sequence homology with previously characterized sRNAs or from a deep RNA sequencing dataset. Intergenic regions (IGRs) of both DNA strands, with 50 nt removed at both ends to reduce false positives due to UTRs from adjacent genes, were extracted from the *E*. *faecium* Aus0004 genome with R scripts and gene annotation files. In a first curation step, IGRs with an HTSeq count <15 and an RPKM normalization <2 were discarded. In a second curation step, sRNA candidates (with adjusted coordinates obtained from both DETR’PROK and read mapping visualization) with an HTSeq count <15 and an RPKM normalization <3 were discarded. Finally, repeated sequences were identified by BlastN and removed during a final curation step. SRD, Staphylococcal regulatory RNA database (srd.genouest.org). BSRD, Bacterial small regulatory RNA Database (http://kwanlab.bio.cuhk.edu.hk/BSRD/); RPKM, Reads per kilobase per million mapped reads.

Thus far, no sRNA has been described in *E*. *faecium*. To investigate the presence of these molecules in this species, we studied the *E*. *faecium* Aus0004 reference strain, a *vanB*-positive CC17 clinical isolate recovered from a bloodstream infection in Australia^27^. This strain, containing a 2.9-Mb circular chromosome and three plasmids, is the first complete *E*. *faecium* genome to be sequenced^27^. This investigation used three different approaches: (*i*) the search for sRNAs already characterized in other bacteria by comparative genomics or from deep RNA-seq of *E*. *faecium* Aus0004, followed by the use of either (*ii*) the HTSeq/DESeq pipeline^28, 29^ or (*iii*) the DETR’PROK workflow^30^ (Fig. 1).

Seven sRNAs were identified by comparative sequence analysis with other gram-positive bacteria. Four of them shared similarities with *cis*-encoded riboswitches, while the others are related to the RNaseP RNA moiety or to tmRNA or 6 S RNA (Table 1). Using RNA-seq data collected from our transcriptomic analysis (see materials and methods), we obtained an initial set of 1,275 srna gene candidates, namely 1,035 from the HTSeq/DESeq pipeline and 240 from the DETR’PROK workflow (Fig. 1). From that set, we kept the candidates detected by both approaches and visualized their mapping patterns and adjacent environments using CLC Genomics, to discard untranslated regions (UTRs). This allowed us to reduce the list to 249 srna gene candidates. As shown in Fig. 1, we applied additional criteria to remove repeated sequences and candidates with weak expression profiles as described^31^. The nucleotide sequence of any candidate identified 10 times or more elsewhere in the Aus0004 genome was systematically discarded. To eliminate weakly expressed transcripts, some of which may have been due to background noise, we applied a stringent cut-off value (mean normalized count ≥10, with the DESeq package). This shortened the list to 54 sRNAs, which, together with the seven sequences retrieved by comparative genomics, produced a total of 61 candidates predicted to be sRNAs in *E*. *faecium* Aus0004. We used the DETR’PROK workflow to infer their nucleotide lengths from the RNA-seq data (Fig. 1 and Table 1).

**Table 1:** List of 61 candidate srna gene candidates expressed by *E*. *faecium* Aus0004.

Gene ID^a^	Start	End	Predicted size	Strand	Mean normalized count^b^
**Putative_sRNA_0030**	**65197**	**65361**	**165**	**+**	**6781**
Putative_sRNA_0040	72553	72861	309	−	123
Putative_sRNA_0070	178719	178943	225	+	199
Putative_sRNA_0080	178780	178948	169	−	48
Putative_sRNA_0110	203399	203733	335	+	716
**Putative_sRNA_0120**	**206313**	**206486**	**174**	**+**	**8721**
**Putative_sRNA_0160**	**231297**	**231666**	**370**	**−**	**147750**
Putative_sRNA_0170	231396	231759	364	+	95
Putative_sRNA_0230	291831	292095	265	+	83
Putative_sRNA_0240	291888	292046	159	−	656
**Putative_sRNA_0280**	**294101**	**294392**	**292**	**+**	**77498**
Putative_sRNA_0290	294178	294318	141	−	38
Putative_sRNA_0300	296905	297012	108	−	33
Putative_sRNA_0410	424232	424336	105	−	34
Putative_sRNA_0430	429269	429402	134	−	30
Putative_sRNA_0440	429270	429377	108	+	49
Putative_sRNA_0510	493298	493357	60	−	55
Putative_sRNA_0560	524176	524527	352	−	69
*sefa2424*.*1T*-*box*	577463	577717	255	+	2709
Putative_sRNA_0620	604168	604657	490	+	820
Putative_sRNA_0670	631417	631660	244	+	628
Putative_sRNA_0690	642761	643120	360	+	565
*sefa2004*.*1T-box*	647190	647261	72	+	83
Putative_sRNA_0750	659145	659228	84	+	326
Putative_sRNA_0860	726327	726431	105	+	227
Putative_sRNA_0870	739675	739963	289	+	496
Putative_sRNA_0880	739774	739966	193	−	45
Putative_sRNA_0940	971099	971202	104	−	173
*sefa1356*.*1T-box*	1002994	1003090	97	−	329
*RNAse P*	1015123	1015380	258	−	835915
Putative_sRNA_0980	1080881	1081114	234	−	286
Putative_sRNA_1030	1154492	1154749	258	−	118
Putative_sRNA_1040	1158983	1159112	130	−	81
Putative_sRNA_1060	1175299	1175386	88	−	57
*sefa1496*.*1FMN*	1246361	1246484	124	−	401
Putative_sRNA_1150	1275233	1275376	144	−	502
Putative_sRNA_1160	1288073	1288157	85	+	177
Putative_sRNA_1180	1325473	1325704	232	−	91
Putative_sRNA_1190	1344161	1344295	135	−	1001
Putative_sRNA_1210	1496538	1496974	437	+	146
Putative_sRNA_1230	1509156	1509447	292	+	178
Putative_sRNA_1240	1509735	1510053	319	+	63
**Putative_sRNA_1260**	**1532660**	**1532783**	**124**	**+**	**2672**
**Putative_sRNA_1300**	**1613157**	**1613386**	**230**	**−**	**64411**
Putative_sRNA_1420	1802650	1802750	101	+	59
***tmRNA***	**1820183**	**1820548**	**366**	**+**	**1313639**
Putative_sRNA_1520	1914911	1915066	156	−	97
Putative_sRNA_1530	1915341	1915549	209	+	86
Putative_sRNA_1900	2327902	2328162	261	−	247
Putative_sRNA_1930	2399192	2399296	105	+	143
Putative_sRNA_1940	2399408	2399705	298	−	244
Putative_sRNA_2020	2497479	2497545	67	+	167
**Putative_sRNA_2050**	**2519586**	**2519703**	**118**	**−**	**551**
*6 S RNA*	2529629	2529815	187	+	759565
Putative_sRNA_2190	2688097	2688534	438	+	175
Putative_sRNA_2200	2688252	2688379	128	−	45
**Putative_sRNA_2210**	**2696000**	**2696387**	**388**	**−**	**1269**
Putative_sRNA_2230	2734869	2735007	139	−	695
Putative_sRNA_2290	2773987	2774147	161	+	10
Putative_sRNA_2300	2774163	2774493	331	−	285
**Putative_sRNA_2410**	**2907958**	**2908152**	**195**	**+**	**647**

^a^sRNAs detected by comparative genomic analysis are indicated in italics; sRNAs validated by Northern blot are indicated in bold; sRNAs harbored by mobile genetic elements (according to the annotation by Lam *et al*.^27^) are underlined.

^b^Calculated with the DESeq package.

### E. faecium sRNA conservation and experimental validation

**Figure 2:**
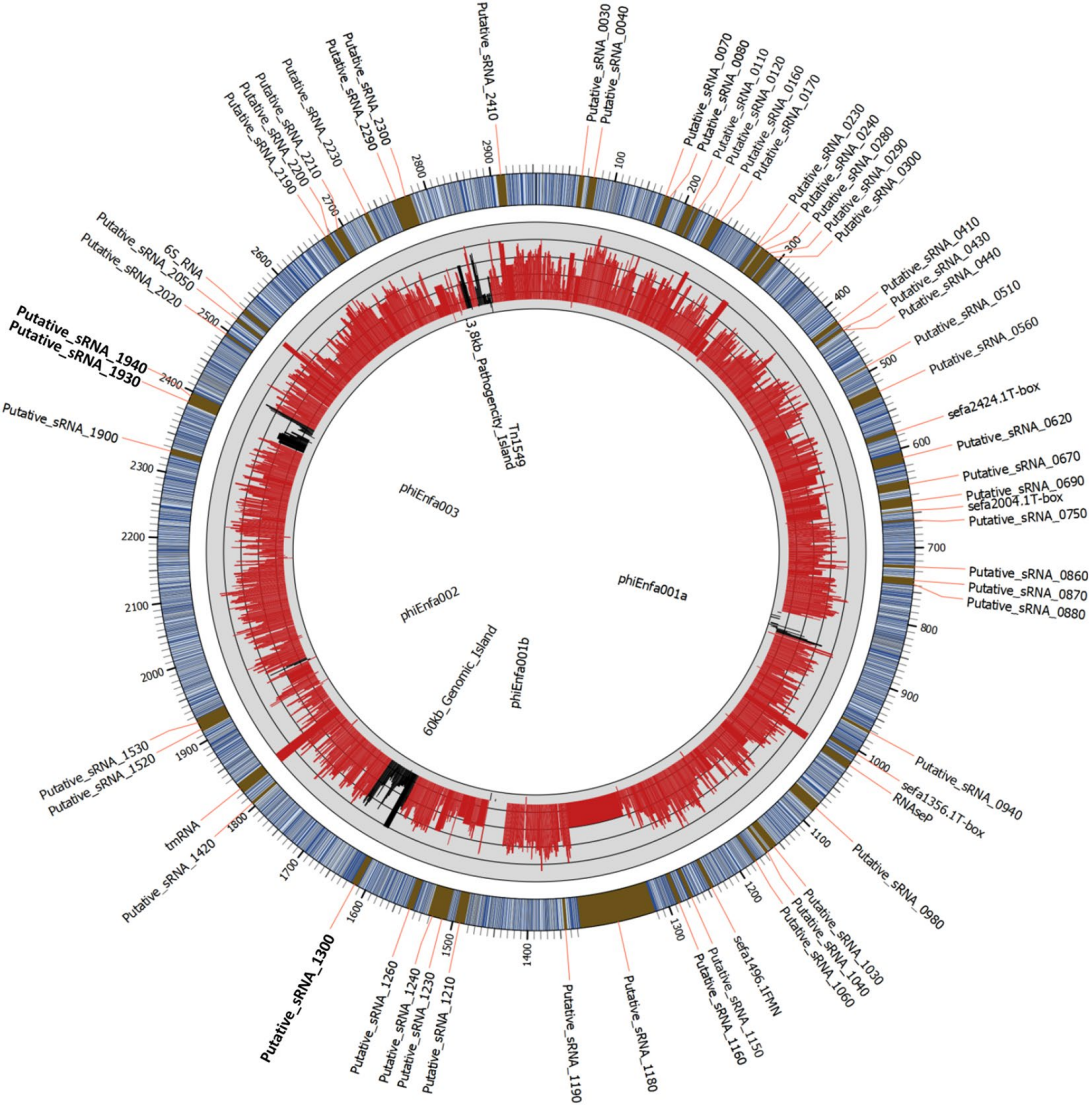
RNA-seq *E*. *faecium* genome annotation discovers 61 new expressed transcripts. The mean normalized count, calculated with the DESeq package, of gene expression is indicated as a black (prophages and Genomic Islands) or red (rest of the genome) line in the gray circle, and thin gray circular lines represent the mean expression level of each gene as a Log_2_ value (Table S3). The outermost circle represents the full *E*. *faecium* Aus0004 genome with a 30-fold magnification of the 61 candidate sRNAs. Candidate sRNAs_1300, _1930 and _1940 (in bold) are located in prophages and Genomic Islands according to the annotation by Lam *et al*.^27^.

We then analyzed the 61 sRNAs initially identified. Figure 2 depicts the genomic location of these transcripts. Note that three srna genes were part of prophages or genomic islands, according to the annotation performed by Lam *et al*.^27^: two (sRNA_1930 and sRNA_1940) in prophage phiEnfa003, and one (sRNA_1300) in an annotated 60-kb genomic island (Fig. 2).

**Figure 3:**
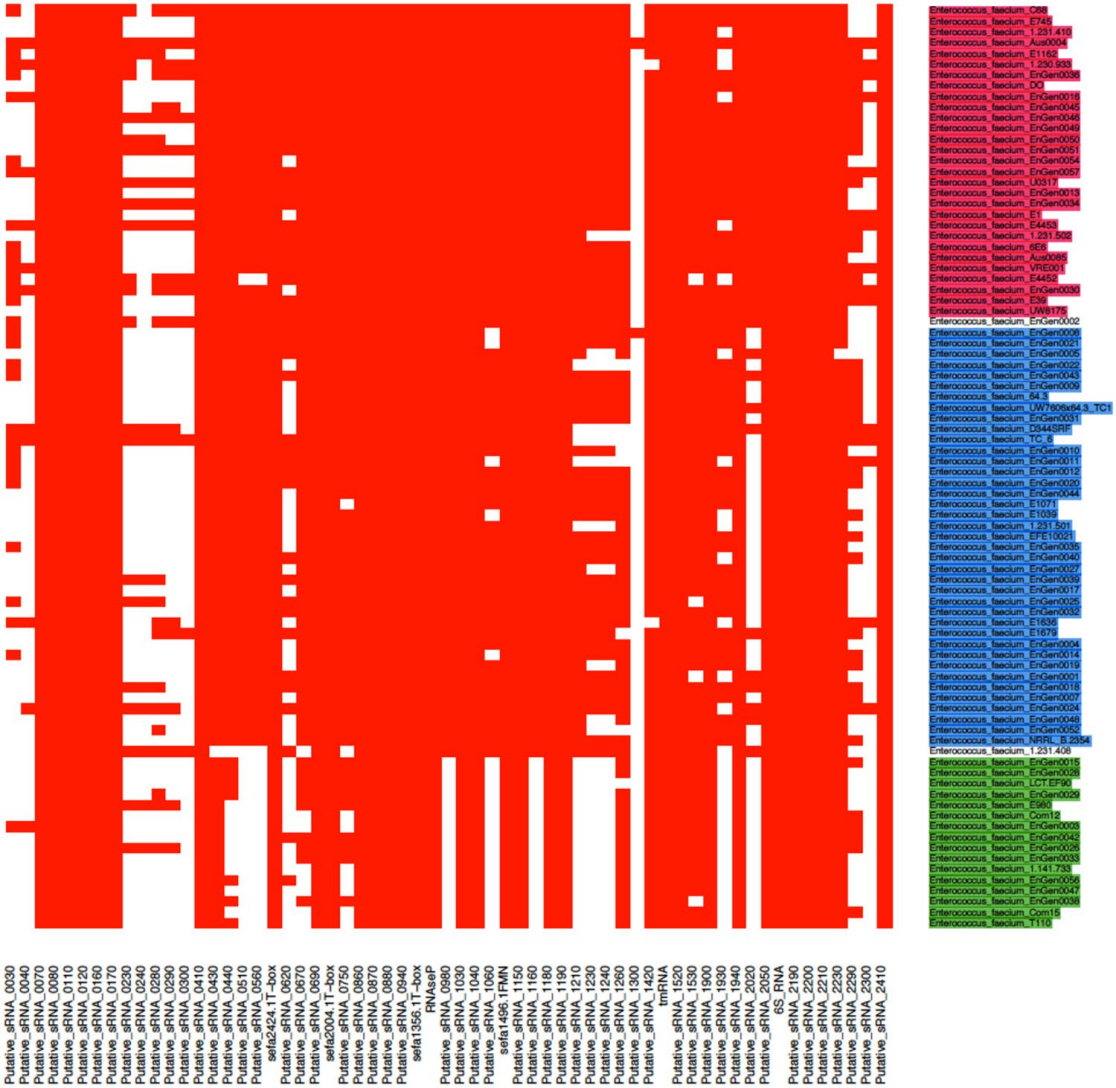
Comparative analysis of the presence and absence of 61 novel sRNAs candidates in *E faecium* strains. A heat map was generated based on the presence (red) and absence (white) of 61 expressed srna genes in the 86 fully sequenced *E*. *faecium* strains that belong to the different phylogenetic clades. Clades A1, A2 and B (according to^7^) are indicated in magenta, blue and green, respectively.

The conservation of these 61 sRNAs candidates among 86 fully *E*. *faecium* sequenced genomes was examined (Fig. 3). Importantly, three clades were identified within the species *E*. *faecium*, (clades A1, A2 and B) as inferred by comparative genomics^7, 32^. Out of the 61 srna candidates, 32 (52%) were conserved among all *E*. *faecium* strains while 13 were absent in clade B strains (Supplementary Table S1). No gene was uniquely found either in clades A1 or A2 while none was specific to Aus0004 (Supplementary Table S1). Of interest, the median number of srna gene candidates was significantly higher in clade A1 strains than in clades A2 and B (*P* < 0.0001) (Supplementary Fig. S1).

**Figure 4:**
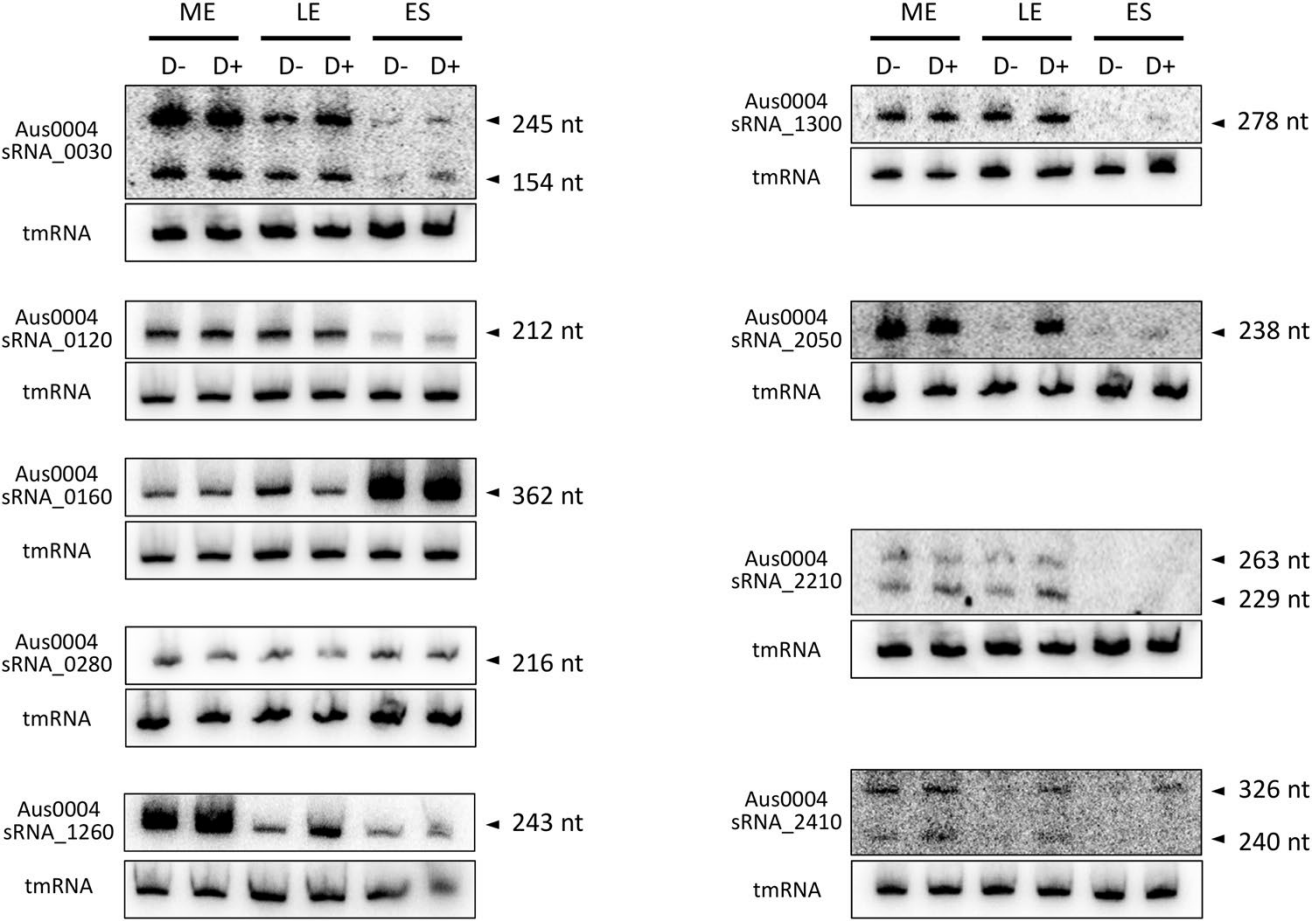
Experimental validation of 10 sRNAs expressed from *E*. *faecium* Aus0004 and the impact of daptomycin SIC on their expression. Northern blots were performed on RNAs extracted from cells collected at an OD_600 nm_ of 0.7, 1.6, and 1.9 corresponding to mid (ME), late (LE) exponential and early stationary (ES) phases of growth. The RNAs were extracted on cells grown in the absence (D−) or presence (D+) of daptomycin SIC. tmRNA levels were used as internal loading controls. The data show a representative experiment among three independent biological replicates.

Analysis of sRNA expression further strengthened our transcriptomic analysis. Ten of the 61 sRNA candidates, selected mainly based on their high levels of expression, inferred from the RNA-seq data (Table 1), were challenged experimentally by Northern blots, including tmRNA (*ssrA*) (subsequently used as an internal control) (Fig. 4). This technique enabled us to monitor the expression of these 10 sRNAs at three time points (ME, mid-exponential; LE, late-exponential; ES, early-stationary) during bacterial growth (Fig. 4). Almost all were expressed at the ME and LE phases, while three (sRNA_1300, sRNA_2050 and sRNA_2210) were not detected at the ES phase (Fig. 4). The nucleotide length of each sRNA was also estimated, based on their PAGE migration, combined with the use of a pre-stain RNA ladder (75 and 100 nt) mixed with tmRNA (366 nt). An excellent match was observed between the data obtained by Northern blots and the predicted sizes based on the RNA-seq data. Three expressed sRNAs (sRNA_0030, sRNA_2210, and sRNA_2410) were produced as two transcripts (Fig. 4).

### Genome-wide transcriptomic analysis after exposure to daptomycin subinhibitory concentrations

Our search for sRNAs expressed by *E*. *faecium* included a global transcriptomic analysis. Using RNA-seq, we compared the transcriptome, including both mRNAs and sRNAs of *E*. *faecium* Aus0004 cultured with ( + Dap) or without (-Dap) a SIC (0.5 μg/ml corresponding to 1/4 MIC) of daptomycin, an antibiotic commonly used to treat VREF infections.

**Figure 5:**
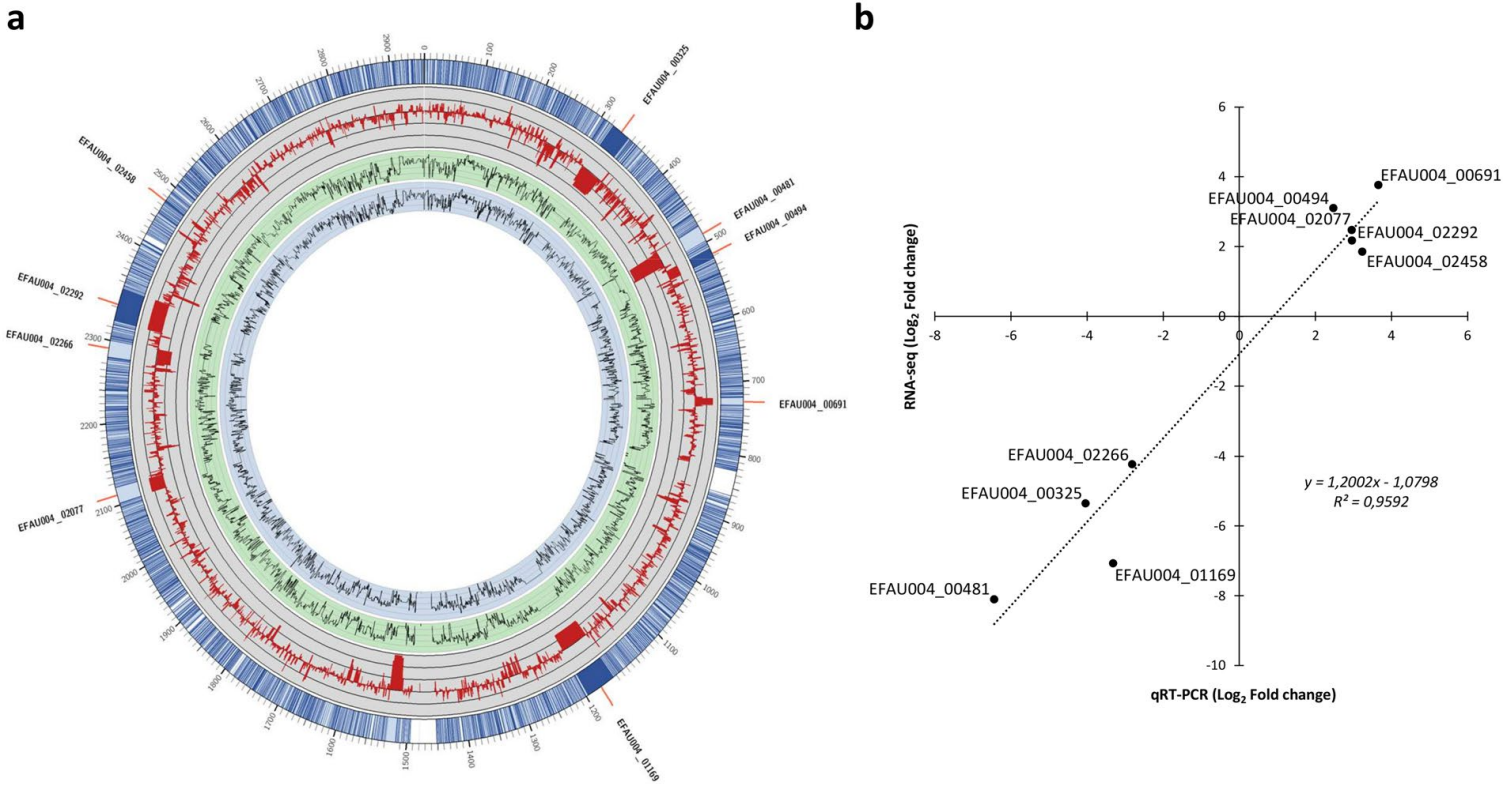
Genome-wide transcriptional response of *E*. *faecium* Aus0004 to daptomycin SIC. (**a**) Global analysis of transcript levels in *E*. *faecium* Aus0004 by RNA-seq. Conditions and ‘+Dap’ and ‘−Dap’ refer to bacterial growth in the presence or absence of daptomycin (concentration at 0.5 μg/mL), respectively. Blue and green inner circles correspond to the mean expression of each gene, as calculated by DESeq, in bacteria grown under ‘−Dap’ and ‘+Dap’ conditions, respectively. The red line in the gray circle represents the baseline, and thin gray circular lines represent four-fold (or log_2_ = 2) changes in expression of each gene (Supplementary Table S3). The outermost circle represents the full *E*. *faecium* Aus0004 genome with a 20-fold magnification of the genes for which expression was confirmed by qRT-PCR. (**b**) Validation of the RNA-seq data by qRT-PCR for 10 selected genes. Mean log_2_ ratios of values determined in the qRT-PCR experiments are plotted against the mean log_2_ ratios of the RNA-seq experiments.

Between 23 and 33 million reads were obtained for each stranded cDNA library made from total RNAs collected at the LE growth phase; 54–75% mapped to the genome of *E*. *faecium* Aus0004, corresponding to average genome coverage of 255 to 417 (Supplementary Table S2). Note that rRNA depletion was highly efficient, with only 1 to 9% of reads corresponding to rRNA genes (Supplementary Table S2). The reproducibility of experimental duplicates was very satisfactory in both conditions (r^2^ =  > 0.97, Supplementary Fig. S2). Between 38 and 72% of reads mapped to coding sequences (CDSs), and 1 to 6 million reads to sRNA candidates (Supplementary Table S2). Experimental challenge by RT-qPCR of gene expression variations detected by RNA-seq, with a Pearson correlation coefficient of 0.9592 (Fig. 5).

#### Overall transcriptome picture

Figure 5 and Supplementary Table S3A present the transcriptome expression levels of cells grown under −Dap and +Dap conditions. Under daptomycin SIC exposure, 280 genes presented significantly altered transcript levels (fold change of expression <−4 or >4, adjusted *P*-value < 0.05), including 80 upregulated and 180 downregulated genes (Supplementary Table S3B and C). Among these 280 genes, 7 (3 downregulated and 4 upregulated) corresponded to sRNA candidate genes that we identified earlier (Supplementary Tables S3B and C). To interpret the RNA-seq data, we performed a COG (Clusters of Orthologous Groups of proteins) functional categorization to evaluate the impacted metabolic pathways (Supplementary Fig. S3)^33^. In the presence of a subinhibitory daptomycin concentration, we observed significant induction of genes coding for proteins involved in nucleotide metabolism and transport, transcription, replication and repair, and cell wall/membrane/envelope biogenesis(Supplementary Fig. S3). Genes coding for proteins involved in energy production and conversion and in carbon metabolism and transport were significantly repressed (Supplementary Fig. S3). Carbon metabolism and transport activity decreased strongly, with 51% (91/180) of the repressed genes belonging to this functional category (Supplementary Table S3C).

#### Differentially expressed mRNAs and sRNAs

The most significant variation of gene expression caused by a subinhibitory daptomycin concentration concerned genes involved in galactose metabolism (EFAU004_00481-EFAU004_00483), with a decrease ranging from −87 to −112 fold (Supplementary Table S3C). This finding suggests that the stress caused by the presence of a low antibiotic concentration deeply modifies the carbon flow, with concomitant alteration of interconnected metabolic pathways and specific use of energy sources available for growth.

Supplementary Table S4 reports the modulation in expression of the genes characterized as related to virulence and antimicrobial resistance^4, 34^ on daptomycin exposure, based on this transcriptomic study. Among the 24 potential virulence genes described in *E*. *faecium* Aus0004, four showed significant change in expression (fold change <−4 or >4, adjusted *P*-value < 0.05), with three genes repressed and only one induced. The *acm* gene, coding for the main collagen-binding adhesin in *E*. *faecium*, was significantly upregulated by a magnitude of 7.8-fold in the presence of daptomycin SIC (Supplementary Table S4). This induction was phenotypically confirmed by collagen-binding assays (Supplementary Fig. S4A), since Acm binds collagen type I and type IV^35^. By contrast, the level of biofilm production did not differ significantly with or without daptomycin (Supplementary Fig. S4B), a finding consistent with the lack of significant change in the expression of the major factors involved in biofilm formation (i.e. *ebpABC*, *empABC* and *esp* ^36–38^) (Supplementary Table S4). Among the repressed genes were two encoded carbohydrate phosphotransferase system (PTS) proteins, *ptsD* and *bepA*, which had fold changes of −7.5 and −10.1, respectively (Supplementary Table S4). Interestingly, both PTS proteins are involved in *E*. *faecium* pathogenesis, that is, absent in human commensal isolates and enriched in isolates responsible for hospital outbreaks and infections^39, 40^. Significant downregulation was also observed in levels of *swpA*, which expresses a protein containing a WxL domain, which in turn plays a role in bile salt stress and endocarditis pathogenesis^41^. No resistance genes showed any significant expression change (fold change <−4 or >4, adjusted *P*-value < 0.05, Supplementary Table S4).

Bacterial sRNAs participate in the regulation of physiological networks and adaptation to specific modifications of environmental conditions, including antibiotic exposure^22^. In addition to the gene-coding variations detected, daptomycin exposure significantly modulated the expression of 7 srna genes, among the 61 candidates (fold change of expression <−4 or >4, adjusted *P*-value < 0.05), upregulating 3 sRNAs (sRNA_0560, +4.9; sRNA_1420, +11.2; sRNA_2290, +6.3) and downregulating 4 (sRNA_0160, −11.2; sRNA_0170, −6.2; sRNA_0290, −5.4; 6 S RNA, −5.9).

The expression of the experimentally validated sRNAs under daptomycin SIC was assessed at three time points during growth by Northern blots (Fig. 4) and qPCRs (Supplementary Fig. S5). These data were compared to those obtained by RNA-seq with RNAs extracted at the LE growth phase and showed satisfactory agreement (Table 2). The transcript level of only one sRNA (sRNA_0160) decreased significantly under antibiotic exposure (fold change of −11.2), and none increased (Table 2). This experimental evidence provides us substantial confidence in the specific responses of this sRNA to daptomycin stress. At the three time points measured during *E*. *faecium* growth under daptomycin exposure, the expression of four sRNAs remained mostly uniform (sRNA_1260, sRNA_1300, sRNA_2050 and sRNA_2410), whereas that of five sRNAs (sRNA_0030, sRNA_0120, sRNA_0160, sRNA_0280 and sRNA_2210) fluctuated substantially (Supplementary Fig. S5).

**Table 2:** Expression variations of 9 sRNAs from E. faecium under daptomycin SIC.

*E*. *faecium* sRNAs^a^	Start	End	Size	Strand	Mean normalized count (RNAseq)		Fold Change^b^ (RNAseq/qPCR)	Adjusted *P*-value
					−Dap	+Dap		
sRNA_0030	65197	65361	165	+	8899	4663	−1.9/−0.2	0.5323
sRNA_0120	206313	206486	174	+	13141	4301	−3.1/−3.0	0.0223
**sRNA_0160**	**231297**	**231666**	**370**	**−**	**271255**	**24245**	**−11.2/−5.9**	**0.0145**
sRNA_0280	294101	294392	292	+	134706	20289	−6.6/−2.6	0.0509
sRNA_1260	1532660	1532783	124	+	1753	3590	−2.0/2.9	0.3698
sRNA_1300	1613157	1613386	230	−	102719	26104	−3.9/−0.7	0.1502
sRNA_2050	2519586	2519703	118	−	403	699	1.7/2.9	0.2385
sRNA_2210	2696000	2696387	388	−	1840	697	−2.6/−1.6	0.3399
sRNA_2410	2907958	2908152	195	+	395	898	2.3/4.1	0.0335

^a^sRNA with significantly altered expression in the presence of daptomycin SIC are emphasized (bold).

^b^The combined data from RNA-seq and qPCR were inferred from RNAs extracted at the late exponential (LE) phase of growth.

### Potential roles of experimentally validated sRNAs in daptomycin resistance

**Figure 6:**
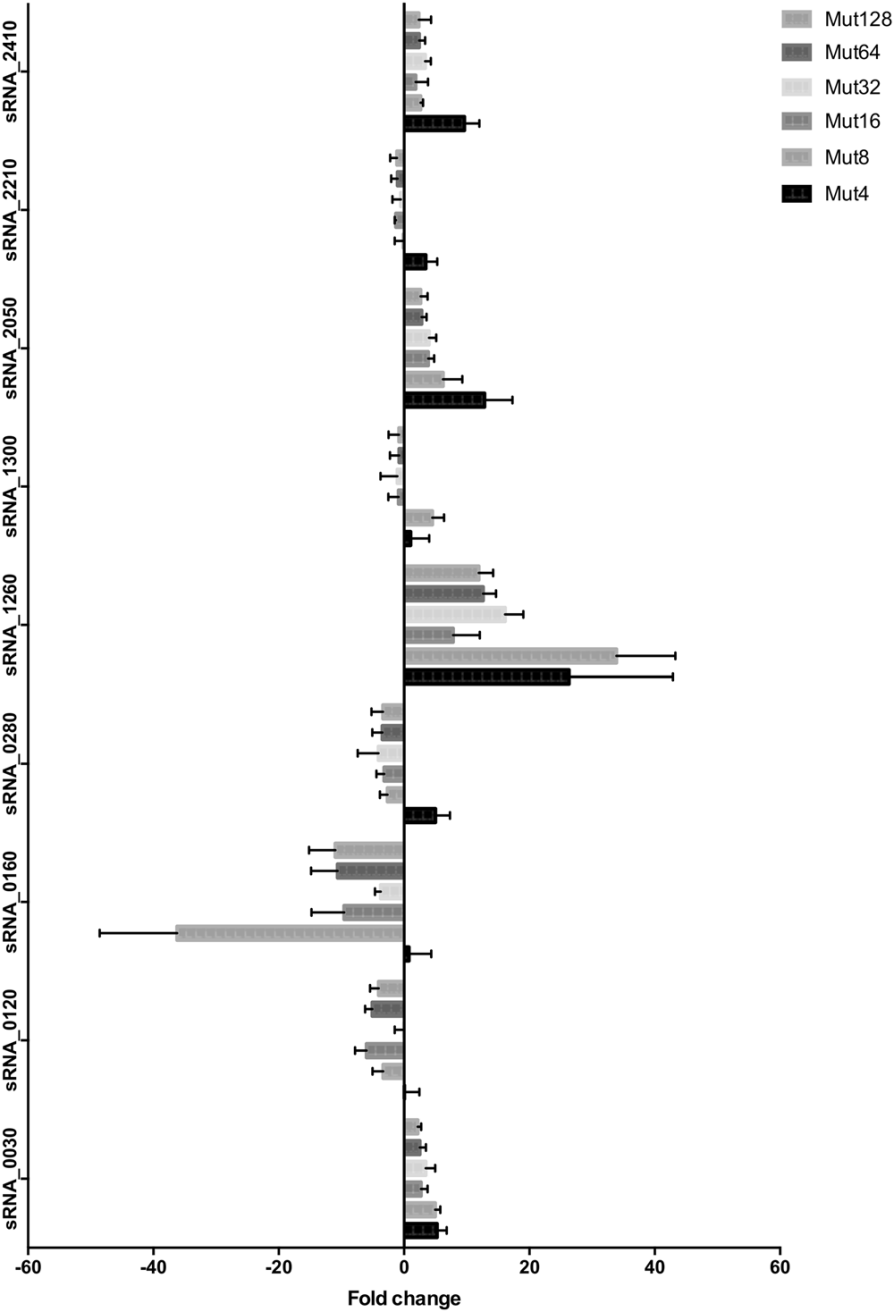
sRNA expression in isogenic stepwise daptomycin-resistant mutants of *E*. *faecium* Aus0004. A series of *in vitro* mutants derived from strain *E*. *faecium* Aus0004 with increasing daptomycin MICs (4, 8, 18, 32, 64, and 128 μg/mL *vs*. 2 μg/mL for the parental strain)^17^ was tested. qRT-PCR experiments were performed in triplicates on RNAs extracted from cells collected at an OD_600 nm_ of 1.6 (late exponential growth phase).

Further experiments used a series of incremental daptomycin-resistant mutants (named Mut4 to Mut128) to investigate the link between the expression of the nine experimentally validated sRNAs (all except tmRNA) and *E*. *faecium* daptomycin resistance. These mutants were previously obtained *in vitro* from *E*. *faecium* strain Aus0004 and have daptomycin MICs ranging from between 4 to 128 mg/L, while the MIC of the parental strain was 2 mg/L^17^. After extracting total RNAs at the LE phase, we studied the expression of these nine sRNAs with qPCR. Expression of three sRNAs (sRNA_0160, sRNA_1260, sRNA_2050) was modified sharply in the stepwise daptomycin-resistant mutants (Fig. 6), with sRNA_0160 repressed in Mut8 to Mut128 (fold changes from −36 to −4), sRNA_1260 (fold changes from + 8 to + 36), and sRNA_2050 (fold changes from + 3 to + 13) induced over the series (Fig. 6). This finding suggests that these sRNAs play a role in the progressive acquisition of daptomycin resistance by *E*. *faecium*.

## Discussion

Many studies have identified sRNAs in a multitude of bacterial genomes, but mainly in gram-negative bacteria. In recent years, however, large numbers of sRNAs have been identified in a dozen gram-positive bacteria^42^, including *E*. *faecalis* ^24–26, 43^. Enterococci are highly adapted members of the intestinal microbiota of a range of hosts spanning the animal kingdom^8^. They are also leading opportunistic hospital pathogens that cause infections often resistant to many therapeutic options.

Two species, *E*. *faecalis* and *E*. *faecium*, cause the vast majority of hospital-acquired enterococcal infections in humans^44^. Unfortunately, the knowledge acquired about the sRNome of *E*. *faecalis* cannot be translated to *E*. *faecium* since these two species are at opposite ends of the phylogenetic tree^8^: *E*. *faecalis* occurs in one of the oldest branches of the genus, whereas *E*. *faecium* arose more recently. Of these two, it is *E*. *faecium* that has most often acquired resistance to several classes of antibiotics, including vancomycin^45^. In fact, epidemic hospital-adapted CC17 strains, such as *E*. *faecium* Aus0004, are part of a human hospital-adapted lineage (clade A1) that emerged approximately 75 years ago from the animal-associated lineage (clade A2) after the introduction of antibiotics: both differ genetically from the human community-associated lineage (clade B)^7^. These successful hospital-adapted strains have the ability to acquire adaptive elements cumulatively by horizontal gene transfer, a feature known as “genetic capitalism” and associated with the emergence of high-risk MDR clones^46^. The larger genome observed in clade A1 strains compared with those in clades A2 and B^7^ suggests that they harbor more coding genes. Even if the experimental validation of all 61 candidate genes would be necessary, our data suggest that it is also likely true for the non-coding sRNA genes. Indeed, strains from clade B harbor much less srna genes (median number = 39) than strains belonging to clades A1 (median number = 54) and clade A2 (median number = 50).

Bioinformatic tools combined with experimental analyses enabled us to identify 61 sRNA candidates from the genome of *E*. *faecium* Aus0004, a clade A1 strain. These srna genes are scattered throughout the entire bacterial chromosome, including in pathogenicity islands. We are aware that this pioneering study was performed only in one strain, a *vanB* VRE clinical isolate. We should extend our investigations in other *E*. *faecium* genetic backgrounds that may reveal discrepancies in their overall sRNA contents. Two thirds of them were detected in all of the *E*. *faecium* strains that have so far been fully sequenced. Most of these srna genes (excluding tmRNA, RNase P RNA and sRNA_0030), however, have no sequence homologs in *E*. *faecalis*. This finding may imply that enterococci possess their own set of sRNAs, as observed for *S*. *aureus* and other staphylococci^47^. Their predicted lengths, ranging from 67 to 437 nts (Table 1), are compatible with their being sRNAs^48^. As suspected, these bacterial species express tmRNA, which is required for ribosome rescue during translation of faulty mRNAs^49^, RNase P RNA for 5′-end maturation of tRNAs^50^, and 6S RNA, which interacts with the primary holoenzyme form of RNA polymerase to influence transcription^51^. Strikingly, 6S RNA expression decreases 6-fold on daptomycin exposure; the biological link thus revealed between 6S RNA and antibiotic response in this bacterial strain requires further investigation. Also of interest is sRNA_0030, whose nucleotide sequence was identified, with an identical nucleotide sequence, in different firmicutes (*Streptococcus*, *Lactobacillus*, *Staphylococcus*, *Clostridium* and *Listeria*), as well as plasmids from gram-negative bacteria (*Neisseria gonorrhoeae* and *Escherichia coli*) and mycoplasmas (*Ureaplasma urealyticum*). sRNA_0030 is expressed from an integrative transposon of the Tn*916* family carrying the *tet*(M) gene, which confers tetracycline resistance^52, 53^. It is expressed 30 to 50 nts upstream from the initiation codon of *tet*(M) in various strains from the firmicutes bacterial phylum, including *S*. *aureus*, suggesting that it may be involved into regulation of tetracycline resistance, with no implications into daptomycin exposure and resistance.

An in-depth expression study was carried out on the most expressed sRNAs that were confirmed by three independent experimental evidences including RNA-seq, qPCR and Northern blots. All sRNAs possess specific expression profiles during *E*. *faecium* growth, some accumulating early on (sRNA_1260), or at later growth stages (sRNA_0160), reminiscent with their regulatory functions.

Daptomycin is a lipopeptide antibiotic with bactericidal activity against gram-positive bacteria, including multidrug-resistant nosocomial pathogens such as methicillin-resistant *S*. *aureus* (MRSA) and VRE^54^. It inserts into the bacterial cell membrane by a calcium-dependent mechanism^13, 55^ and forms oligomeric pores, which results in ion leakage and membrane potential dissipation^56^. In *E*. *faecium*, daptomycin resistance results from alteration of regulatory systems involved in the bacterial cell envelope stress response, as *liaFSR*, and enzymes involved in phospholipid metabolism, as *cls* ^16^.

Transcriptomic analysis was performed using reference strain *E*. *faecium* Aus0004, a *vanB*-positive clinical isolate belonging to clade A1^27^. This strain has a 2.9-Mb circular chromosome composed of 2,753 ORFs, including several virulence factors such as enterococcal surface protein (*esp*) and collagen-binding adhesin (*acm*). This study has revealed up and down regulations of a small subset of virulence genes in *E*. *faecium* under daptomycin exposure. Indeed, *acm* was significantly upregulated, with a magnitude fold of 7.8 (Supplementary Table S4). Daptomycin-induced expression was phenotypically confirmed by collagen-binding assays (Supplementary Fig. S4A). Recently, we demonstrated such antibiotic-dependent upregulation of *acm* in the presence of ciprofloxacin SIC^12^. Since Acm is a primary collagen adhesin involved in experimental infective endocarditis^57^, *acm* induction caused by antibiotics SICs may be clinically relevant. By contrast, *ptsD* (−7.5-fold change) and *bepA* (−10.1-fold change) virulence genes that contribute to intestinal colonization and endocarditis/biofilm formation, respectively,^39, 40^ were significantly downregulated. Even though *bepA* is implicated in biofilm formation, there was no significant change in biofilm construction in the presence of daptomycin SIC (Supplementary Fig. S4B). However, numerous additional genes are involved in biofilm production and regulation in *E*. *faecium*, such as *ebpABC*/*empABC*, *esp*, *asrR*, *ebrB*, *atlA*_*Efm*_, *sgrA*, *capD* ^35–38, 58–60^, implying that biofilm regulation in this bacterium is a complex and multi-component process, as for many other bacteria^61^. Because several sRNAs are involved in the regulation of biofilm formation in a variety of bacteria^62^, a reasonable hypothesis is that some of the riboregulators described here could also be involved. Using stringent cut-off values for transcriptomic analysis (*i*.*e*. fold change <−4 or > 4 and *P* < 0.05), no resistance genes showed significant change in expression level. However, genes involved in daptomycin resistance, such as *liaFSR* and *cls* ^16^ had some degree of changes in expression (Supplementary Table S4). It is noteworthy since expression level of the *liaFSR* operon is linked to daptomycin resistance levels^17^.

A growing number of sRNAs are implicated in bacterial antibiotic resistance^18^, although physiological and molecular explanations of their involvement is largely unknown. In other gram-positive human pathogens such as *S*. *aureus*, some sRNAs are part of a coordinated transcriptional response to specific antimicrobial exposures^22^, or are involved in glycopeptide resistance^21^. The expression level of one sRNA (sRNA_0160) was significantly downregulated under daptomycin exposure. In addition, sRNA_0160 was also significantly repressed in daptomycin-resistant mutants. The pathways leading to daptomycin-resistance selection *in vitro*, however, may not entirely represent the process that occurs *in vivo* under daptomycin therapy, thus these data may not be conclusive in clinics. Taken together, it suggests that sRNA_0160 would be connected to antibiotic response and resistance in *E*. *faecium*, and therefore further investigations regarding the functions, mechanisms and molecular targets of this riboregulator will be conducted.

### Concluding remarks

Our study demonstrates the existence of sRNAs expressed by *E*. *faecium*, a notorious ESKAPE opportunistic human pathogen. These novel sRNAs could be included, in the future, in a new resource for the hundreds of sRNAs identified in gram-positive bacteria^63^, as recently documented for staphylococci^47^. The 10 most expressed sRNAs expressed by *E*. *faecium* were investigated further. The expression of some sRNAs is induced upon daptomycin SIC exposure, and their possible connections with antibiotic resistance acquisition were identified. The set of daptomycin-responsive genes, including several virulence genes and riboregulators, was identified and some phenotypes validated experimentally. It is anticipated that such a detailed inventory of transcription units and sRNAs will provide substantial assistance for future investigations of this major cause of hospital-acquired human infections worldwide.

## Methods

### Bacterial strains, growth conditions, and antimicrobial susceptibility testing

*E*. *faecium* strain Aus0004^27^ and isogenic daptomycin-resistant mutants^17^ were grown at 37°C in Brain Heart Infusion broth (BHI) or on agar plates (Becton Dickinson). Minimal inhibitory concentrations (MICs) of daptomycin were determined in triplicates on Mueller-Hinton agar with E-test strips (bioMérieux, Marcy l'Etoile, France). The growth kinetics of *E*. *faecium* strain Aus0004 were assessed *in vitro* in BHI broth with increasing (0.06 to 2 µg/ml) daptomycin concentrations at 37°C for a 24-hour period, and the experiments were performed in triplicates. The subinhibitory concentration (SIC) corresponded to the highest antibiotic concentration with no significant effects for bacterial growth.

### RNA-seq analysis and RT-qPCR validation

*E*. *faecium* strain Aus0004 was cultured at 37°C until the late exponential growth phase (OD 1.6) in BHI broth (adjusted to 50 µg/ml Ca^2+^), with and without daptomycin at SIC, and total RNA was extracted with the ZR Fungal/Bacterial RNA Miniprep kit (Zymo Research, Irvine, CA). Residual chromosomal DNA was removed by treating samples with the TURBO DNA-free kit (Life Technologies, Saint Aubin, France). Samples were quantified with a Biospec-Nano spectrophotometer (Shimadzu, Noisiel, France), and their integrity was assessed with the Agilent 2100 bioanalyzer. A Ribo-Zero^TM^ Magnetic kit from gram-Positive Bacteria (Epicentre, France) was used to remove the 23S, 16S and 5S rRNAs from the samples. rRNA depletion was verified on an Agilent 2100 bioanalyzer. The next steps, from mRNA fragmentation to high-throughput sequencing, were performed by ProfileXpert (Lyon, France). The library was constructed with the dUTP-Based NEXTflex™ Directional RNA-Seq Kit, and the samples were sequenced on an Illumina Hi-Seq 2500 platform (single-end, 50 cycles). The experiments were done in duplicates. The COG analysis was performed using updated database^64^. For the reverse transcription-qPCR experiments challenging the differentially expressed mRNAs, cDNA was synthesized from 25 ng total RNA with a QuantiTect RT kit (Qiagen, Courtaboeuf, France). Transcript levels were confirmed by the ΔΔCt method with *adk* as the housekeeping control gene^12^ (Supplementary Table S5). These experiments were performed in triplicates.

### Candidate sRNA identification and conservation analysis

The *E*. *faecium* Aus0004 genome sequence and annotation files were obtained from NCBI at: ftp://ftp.ncbi.nlm.nih.gov/genomes/Bacteria/. Two approaches were used to identify candidate sRNAs in the *E*. *faecium* genome. First, the updated annotation files (in GFF format) including all of the intergenic regions and the antisense portions of the coding genes, were created. The Fastq files were mapped onto the bacterial genomic sequence with BWA^65^. The reads were counted by HTSeq count^28^ with the GFF files. An RPKM normalization procedure was applied, and all the sRNA candidates with an RPKM <3 and HTseq-count <15 were removed. The retained candidates were submitted to Rfam database^66^ and bacterial sRNA databases (BSRD, SRD), which allowed us to identify and keep the initial nomenclature of seven srna genes (sefa2424.1T-box, sefa2004.1T-box, sefa1356.1T-box, RNase P, sefa1496.1FMN, tmRNA, 6S RNA), and we then confirmed this prediction with Infernal^67^. In the second approach, DETR’PROK^30^, a workflow devoted to prokaryotic sRNA identification, was applied to our dataset with the standard annotation downloaded from NCBI. From BWA alignments, DETR’PROK clustered reads located within non-annotated regions of the *E*. *faecium* Aus0004 genome. The workflow was set to retain all clusters containing more than 50 nucleotides, more than 12 reads, and located at least 25 nucleotides apart from any coding sequence as described^31^. The outputs obtained from both methods were compiled, to produce a list of the sRNA candidates detected by both approaches. The criteria of sRNA conservation analysis among the *E*. *faecium* strains was performed using BlastN with 70% identity and 60% sequence coverage.

### Candidate sRNA experimental assessment by Northern blot and qPCR

RNA extractions were performed at three time points during growth – middle exponential (ME, OD 0.7), late exponential (LE, OD 1.6), and early stationary (ES, OD 1.9). Extractions were performed as reported^31^. Cell pellets were dissolved into 500 µL of lysis buffer, and cells were broken by acid-treated glass beads and phenol. Total RNA was extracted by phenol/chloroform and ethanol precipitated overnight. RNA samples (15 μg) were loaded on denaturing 7.5% PAGE and transferred onto Zeta probe GT membranes (Bio-Rad) in 0.5 × TBE. Membranes were hybridized with specific ^32^P-labeled probes in ExpressHyb solution (Clontech, USA), washed, exposed, and scanned onto a PhosphorImager (Molecular Dynamics). For the RT-qPCR experiments, cDNA synthesis was performed with the High-Capacity cDNA Archive Kit (Applied Biosystems, Foster City, CA, USA) and quantitative PCR with the Power Sybr® Green PCR Master Mix (Thermofisher Scientific). Transcript levels were determined by the ΔΔCt method, with *adk* as the control (Supplementary Table S5). Each experiment was performed in triplicates.

### Collagen binding and biofilm formation assays

High-binding microtiter plate wells (Immulon 2 HB, Corning) were coated overnight at 4 °C with collagen at 10 μg/mL (from human fibroblast, Sigma-Aldrich, France) or bovine serum albumin (BSA, Sigma-Aldrich, France) as negative controls. After washing the wells, the remaining protein-binding sites were blocked by 1% PBS-BSA for one hour. Two *E*. *faecium* Aus0004 cultures were grown overnight, one with and one without daptomycin SIC, in BHI adjusted to 50 µg/ml Ca^2+^. Cells were centrifuged and re-suspended into 1 mL PBS; 100 µL of cells (10^8^ CFU/mL) was added to the wells and incubated at 37 °C for 2, 6, and 18 hours. The wells were carefully washed four times at each time point with 100 µL PBS. To recover the *E*. *faecium* cells bound to collagen, the wells were scratched and re-suspended into 100 µL PBS. Serial cell dilutions and inoculations onto BHI agar enabled us to count the adherent bacteria on collagen.

The biofilm formation was measured as previously described^9^. Each assay was repeated three times in at least three independent experiments. Statistical comparison conditions at each time point was performed using the unpaired *t* test.

## Electronic supplementary material

S1

S2

S3

S4

S5

supplemental info
