# Supplementary material for: Small RNAs in vancomycin-resistant Enterococcus faecium involved in daptomycin response and resistance
Source: Sci Rep. 2017 Sep 11;7:11067. doi: 10.1038/s41598-017-11265-2 (PMC5593968; doi:10.1038/s41598-017-11265-2)
Supplement: Supplementary file 6 — supplemental info [file 41598_2017_11265_MOESM6_ESM.pdf]

Small RNAs in vancomycin-resistant *Enterococcus faecium* involved in antibiotic response and resistance

Clara Sinel[1], Yoann Augagneur[2], Mohamed Sassi[2], Julie Bronsard[2], Margherita Cacaci[3], François Guérin[1,4], Maurizio Sanguinetti[3], Pierrick Meignen[5], Vincent Cattoir[1,4,6§*], & Brice Felden[2*]

[1]University of Caen Normandie, EA4655, Caen, France. [2]Inserm U1230-Biochimie pharmaceutique, Rennes University, Rennes, France. [3]Catholic University of Sacred Heart, Institute of Microbiology, Rome, Italy. [4]Caen University Hospital, Department of Clinical Microbiology, Caen, France. [5]University of Caen Normandie, IUT (department "STID"), Caen, France. [6]National Reference Center for Antimicrobial Resistance (lab Enterococci), Caen, France.

[§]**Current address:** Inserm U1230-Biochimie pharmaceutique, Rennes University, Rennes, France

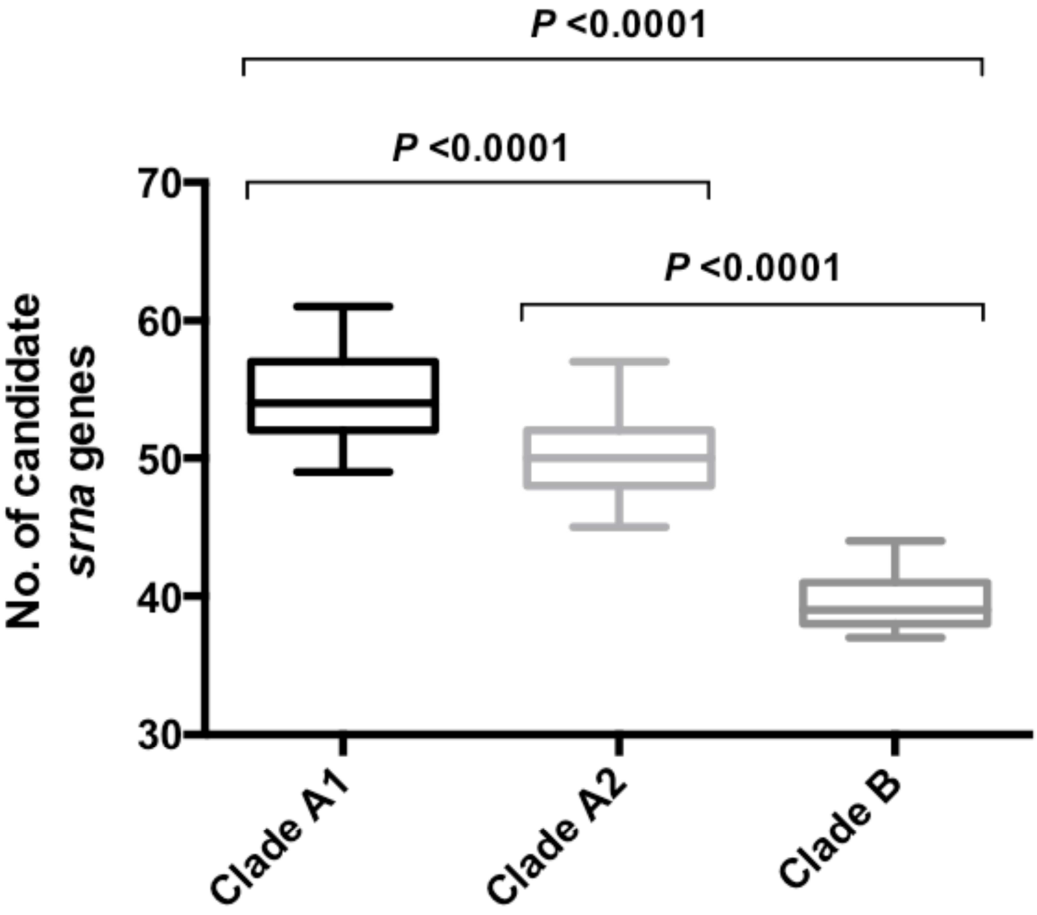

Fig. S1. Number of *srna* genes in strains belonging to the different *E. faecium* phylogenetic clades (A1, A2 and B). Mean values and standard deviations are indicated as well as *P* values (calculated by an unpaired bilateral *t* test).

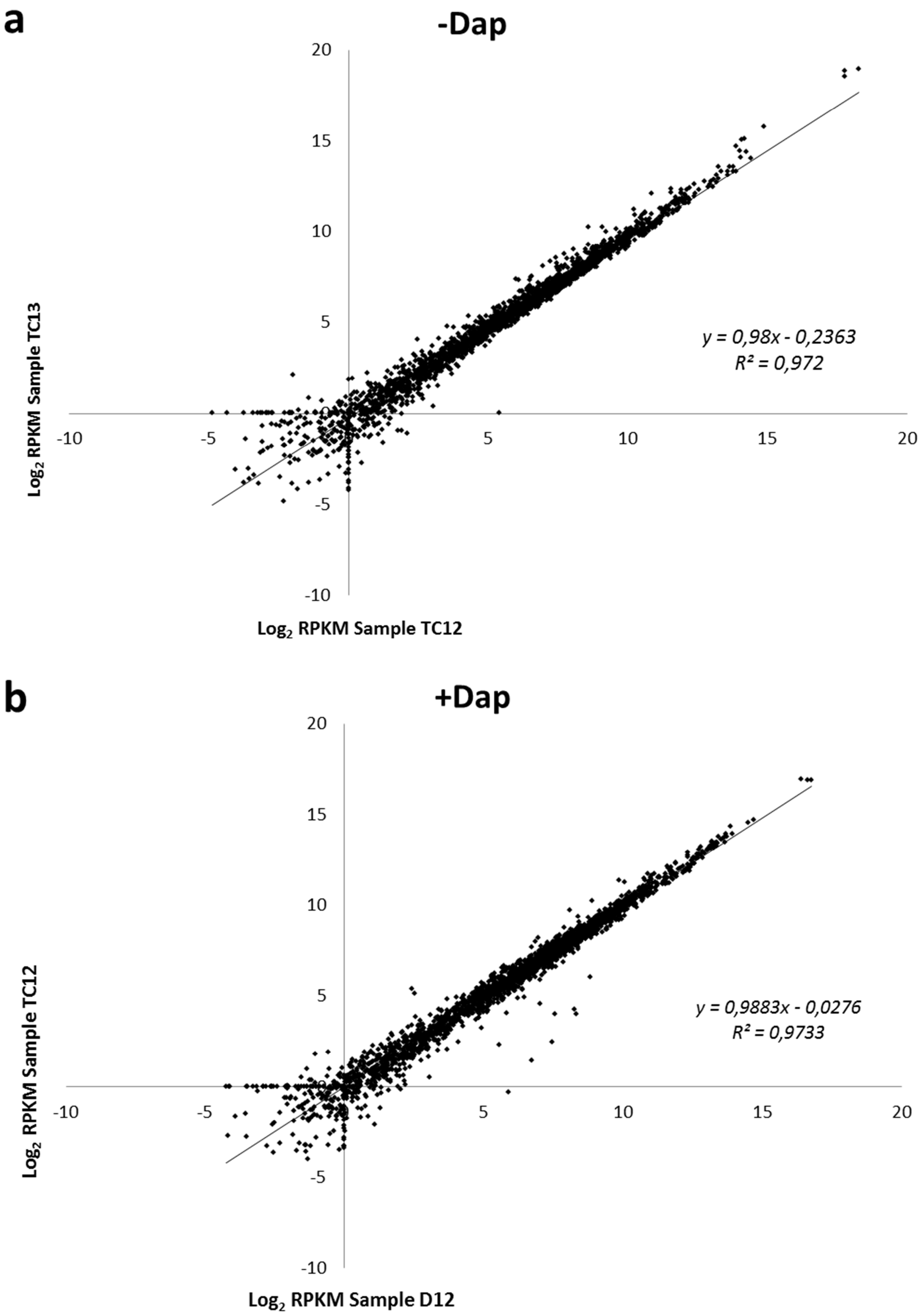

Fig. S2. Reproducibility of duplicate RNA-seq experiments performed in the presence (a) or absence (b) of daptomycin (0.5 μg/mL concentration). The expression of genes in different

RNA-seq samples was compared with normalized RPKM values calculated as follows: (# reads for the gene*10^9) / (# total of reads*size of the gene).

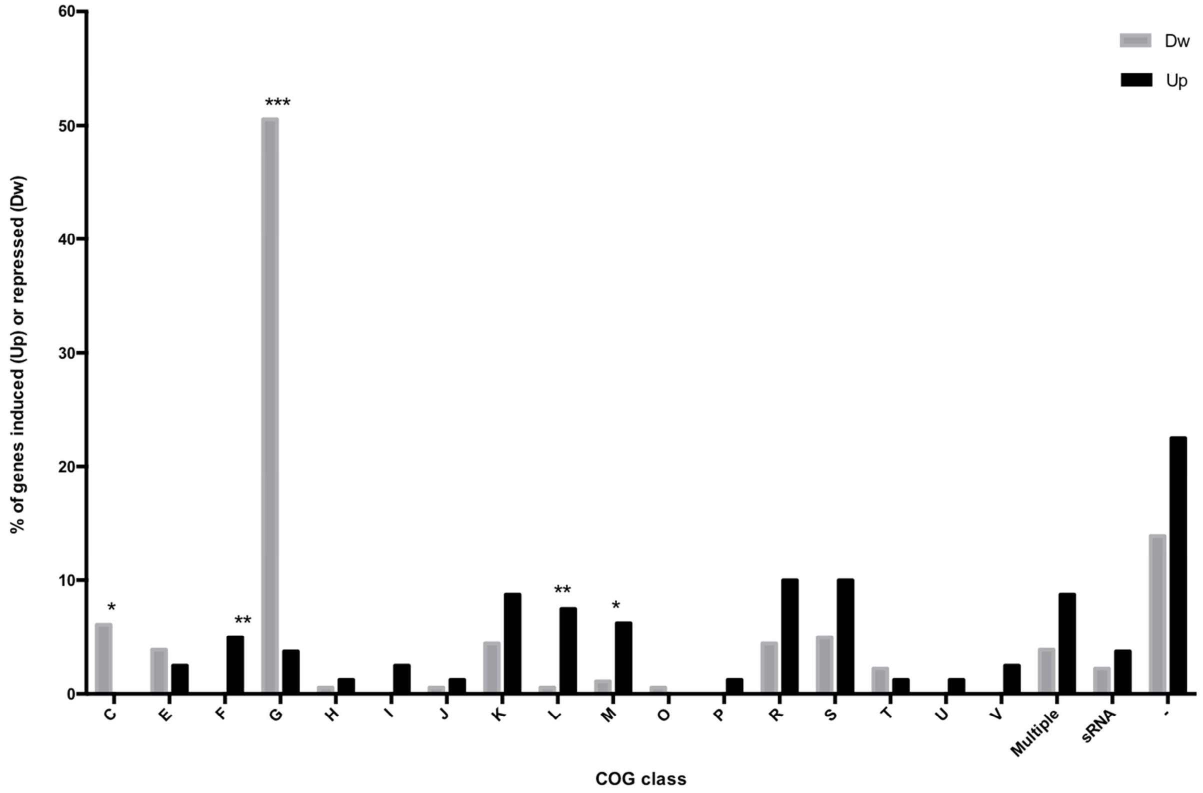

Fig. S3. *E. faecium* genes up- or down-regulated by exposure to daptomycin SIC. Classification in COG classes (see Table S3 for abbreviations) of the 260 genes whose expression is significantly induced (n=80) or repressed (n=180) by daptomycin SIC. Percentages of genes with a change in expression level lower or greater than 2 log_2-folds are represented in gray and black bars, respectively. Statistically significant differences, with Fisher's exact test, are indicated as follows: *, $P<0.05$; **, $P<0.01$; ***, $P<0.001$.

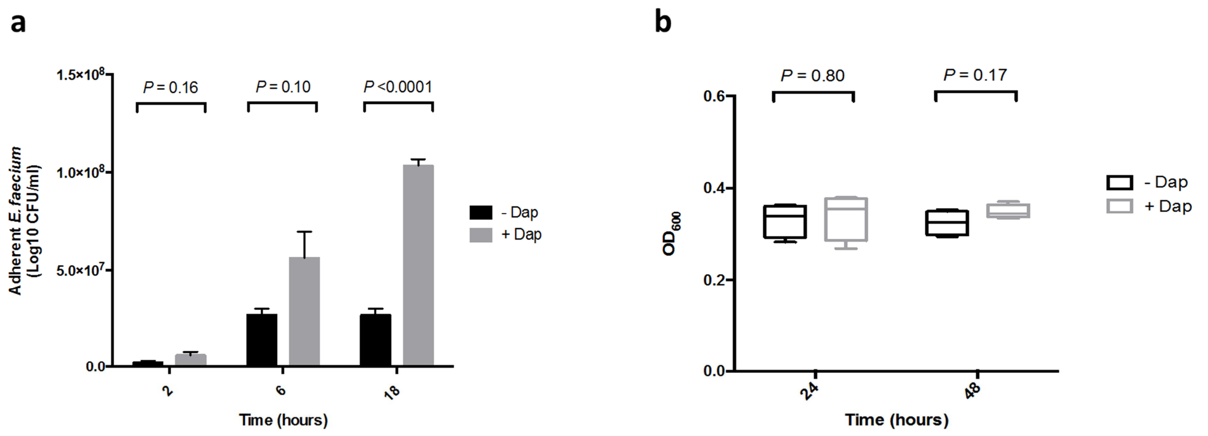

Fig. S4. (a) Adhesion of the *E. faecium* Aus0004 strain to collagen in the presence (+Dap) or absence (-Dap) of daptomycin (concentration of 0.5 µg/mL) at different time points (2, 6, and 18 h). Adherence level is expressed as mean Log_{10} values (± standard deviations) of the number of adherent *E. faecium*. Statistical comparisons between -Dap and +Dap conditions at each time point used the unpaired *t* test, and the corresponding *P* values are indicated. (b) Ability of the strains to form biofilm on a polystyrene surface is shown after 24 and 48 h of incubation. Results are expressed as $OD_{600\ nm}$ measurements and median and interquartile range values are shown. Statistical comparison between -Dap and +Dap conditions at each time point was performed with the unpaired *t* test, and the corresponding *P* values are indicated.

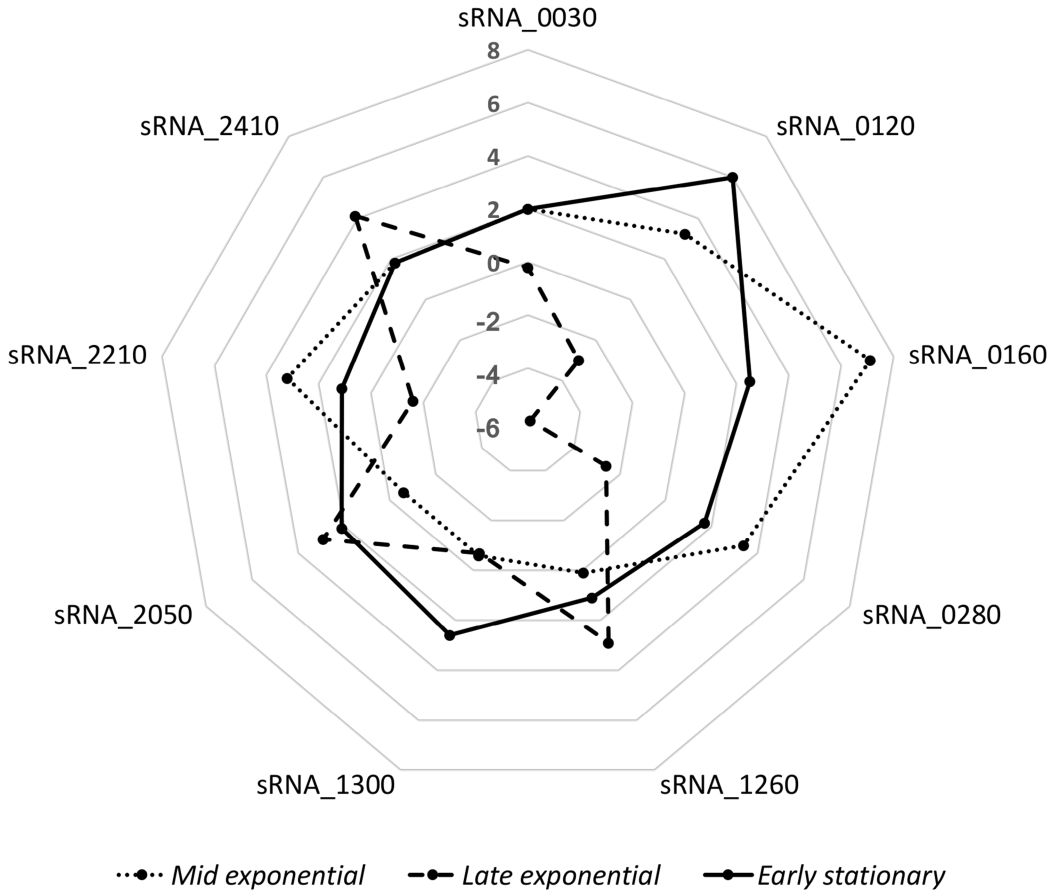

Fig. S5. Switches in *E. faecium* sRNA expression on exposure to daptomycin SIC during growth. qRT-PCR experiments were performed in triplicate on experimentally validated sRNAs extracted from cells collected at an $OD_{600\ nm}$ of 0.7, 1.6, and 1.9 corresponding to mid (ME), late (LE) exponential and early stationary (ES) phases of growth. The nonagons of incremental sizes correspond to the fold changes of sRNA expression induced by daptomycin, from -6 to +8 fold changes in expression levels.
